# Dual-Axis Metasurface Strain Sensor Based on Polarization–Phase-Deformation Relationship

**DOI:** 10.3390/s20051307

**Published:** 2020-02-28

**Authors:** Zhiwei Sun, Run Liu, Hailin Cao, Heling Gong, Mingzhu Du, Shirun Li

**Affiliations:** 1 Chongqing Key Laboratory of Space Information Network and Intelligent Information Fusion, Chongqing University, Chongqing 400044, China; zhwsun@cqu.edu.cn (Z.S.); 20191202038t@cqu.edu.cn (R.L.); lorisgong@163.com (H.G.); 2 State Key Laboratory of Power Transmission Equipment and System Security and New Technology, Chongqing University, Chongqing 400044, China; 3 School of Electronic Science and Engineering, Nanjing University, Nanjing 210023, China; mingzhudoo@163.com; 4 China Academy of Space Technology Xi’an Branch, Xi’an 710100, China; lisrshe@live.cn

**Keywords:** flexible metasurface, strain-sensing, deformation prediction, phase-polarization relationship

## Abstract

Herein, we propose an approach for sensitivity improvement of dual-axis strain sensing using the property of a metasurface (MS) that the phase response shifts sharply with the MS deformation. A feasible approach for phase measurement is first demonstrated by calculating multi-polarized reception when the incident electromagnetic (EM) wave has anisotropic phase values. A flexible MS consisting of periodically arranged lantern-shaped elements is designed and fabricated for dual-axis strain sensing and evaluation based on the proposed method. The simulation and measurement results demonstrated a high sensitivity of the proposed MS for strain sensing in the microwave band. The method can be used potentially in both pressure and tensile sensing. Moreover, the operational frequency can be extended to the THz range and even to the optical band.

## 1. Introduction

In the last decade, metamaterials (MM) have revealed the attractive capabilities for electromagnetic (EM) properties manipulation enabled by artificial structures, and have achieved several exotic and otherwise impossible functionalities such as negative refraction [1,2,3], cloaking [4,5,6], and perfect absorption [7,8,9]. As the two-dimensional simplified form of MM, metasurfaces (MS) [10] have shown unique advantages for the control of the phase, magnitude, and polarization of EM waves, and have been widely applied in wavefront shaping [11,12,13], holographic imaging [14,15,16], and superlensing [17,18,19]. Over the wide frequency range from the microwave to optical bands, MMs and MSs provide an effective and feasible strategy for the manipulation of EM propagation through an appropriate arrangement of meta-atoms that has been a focus of EM research. Recently, strain sensing applications that are challenging for industry have been developed by using stretchable and flexible MMs. The condition of the MMs can be identified through the analysis of the outgoing EM wave from the MMs, and therefore, MMs can be utilized in strain sensing for detection and precise evaluation of the deformation of the employed MS. Following the development of MMs and MSs, various strain sensing applications have been widely used in mechanical, structural, and biological engineering to provide conventional, wireless, and accurate detection.

From the perspective of the operational mechanism, sensing based on the change in the resonance frequency shift [20,21,22,23,24] and the magnitude of transmissivity/reflectivity/absorptivity [25,26,27,28,29,30] with deformation are the two principal methods for strain sensing. Also, some works of chipless radio frequency identification devices (RFID) sensors show a good sensitivity performance, which can be well-referred to sensors design [31,32,33,34]. In [20], numerical equivalent circuit calculations were used to analyze the frequency shift of an MM electric-LC resonator by obtaining its strain-dependent permittivity and permeability. A multilayer array with meta-atoms of strip dipole antenna were designed and fabricated in [28], where a strong response to cross-polarized radiation is modified by breaking the bowtie-shaped junction under the application of strain, resulting in a transmissivity change. Additionally, studies of wrinkled deformation have been performed recently. For instance, in [29], a wrinkled MM layout composed of honeycomb meta-atoms was proposed for strain sensing by analyzing its relative transmission under different stresses. However, even though the sensitivity of the MS strain sensors based on the direct detection of the magnitude or frequency shift has been increasing, the potential of this type of devices for further sensitivity improvement is limited, and the design of these devices becomes more difficult with higher requirements. Thus, the direct detection mechanism has been a bottleneck in MM strain sensing.

In this paper, the relationship between the phase and polarization is investigated to obtain a feasible method for phase measurements that can then provide the basis for determining the relationship between the deformation and reception magnitude of multi-polarized receivers. An approach is proposed for the improvement of the sensitivity of the strain sensor design. The deformation of MS can be sensed and evaluated by analyzing the multi-polarized reception of the reflection from MS under the application of anisotropic strain. An MS sensor composed of periodically arranged lantern-shaped elements is designed, fabricated and characterized to verify the method. Compared with the traditional mechanisms, this approach showed a great enhancement in the sensitivity and can be used in dual-axis strain prediction.

## 2. Phase-Polarization Relationship Analysis

**Figure 1:**
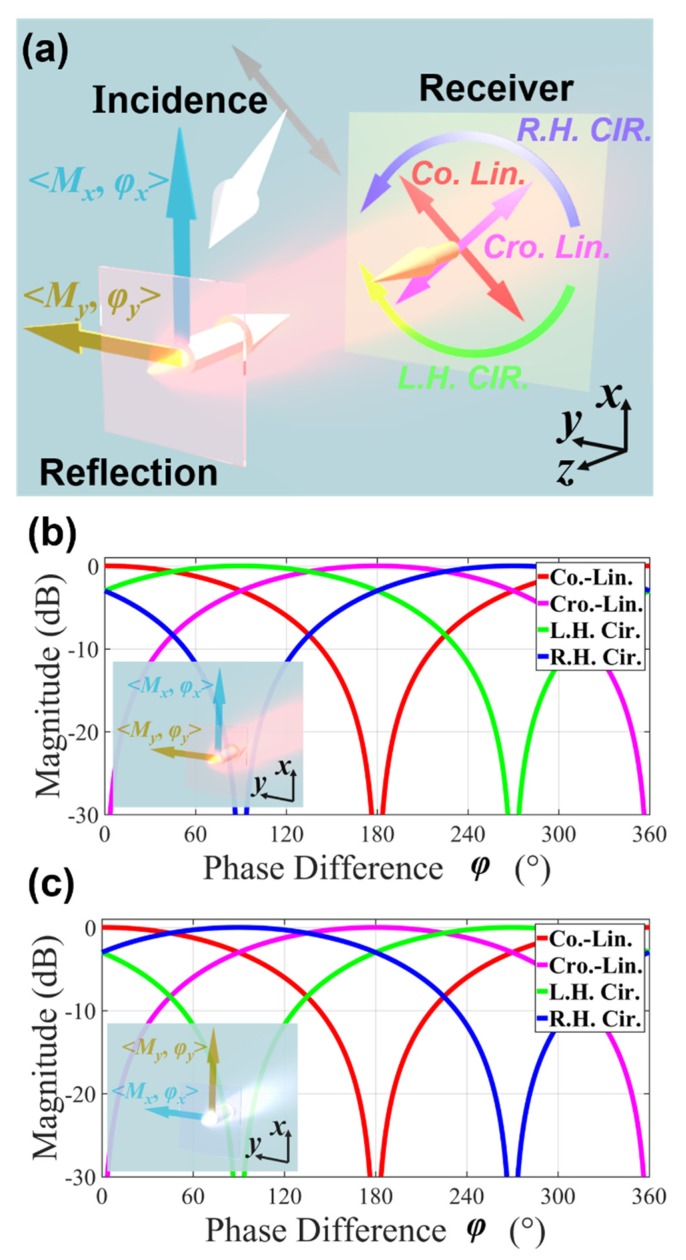
Relationship between the multi-polarized reception of the reflection with an anisotropic magnitude and phase. (**a**) Schematic of the reflection and receiver in the co-, cross linear-polarization and, left-hand, right-hand circular polarization. (**b**) depicts the multi-polarized reception magnitude for the reflective phase states shown in (**a**), while (**c**) shows the multi-polarized reception magnitude in the case of the rotation of the MS by 90°.

First, to provide the theoretical basis for the proposed sensor, the reception performance was investigated for the multiply-polarized receivers, including the co-, cross-, left-hand circular-, and right-hand circular-receivers based on two assumptions: (i) the phase and manipulation of the response of the reflective MS are different along two orthogonal directions (*x* and *y* here), and (ii) the incidence is linear-polarized with the angle between its polarization and the two defined orthogonal directions of reflection equal to 45°, as shown in Figure 1a. In this case, the incidence can be separated into two equal components. Herein, if we normalized the incident wave, the magnitude of the incident EM wave along the *x* and *y* directions are both equal to $\sqrt{2}/2$, with matrix presentation that ${\left[E_{x\_in},E_{y\_in}\right]}^{T}=\frac{\sqrt{2}}{2}{\left[1,1\right]}^{T}$. These conditions are used because the phase shift of a single-polarized EM component cannot attain the variation of the polarization state. By employing Jones Matrix, the reflection process can be described as ${\left[E_{x\_out},E_{y\_out}\right]}^{T}=\left[\begin{array}{c} M_{x}e^{j\varphi_{x}},0\\ 0,M_{y}e^{j\varphi_{y}} \end{array}\right]{\left[1,1\right]}^{T}$. Therefore, the magnitude/phase of the reflection in the *x* and *y* directions are $M_{x}/\varphi_{x}$ and $M_{y}/\varphi_{y}$, respectively. $M_{x}$ and $M_{y}$ should be equal to or less than $\sqrt{2}/2$ where the “less than” case is caused by the absorption of the surface.

As shown in Figure 1a, the reflection is +z direction propagated while the receiver is −z. The magnitude of the receiver under $e_{x}\;M_{x}e^{j\varphi_{x}}+e_{y}\;M_{y}e^{j\varphi_{y}}$ reflection can be calculated as:

(1) $$\{\begin{array}{c} co-lin.:\sqrt{2}/2Re\left[1\cdot \left(M_{x}+M_{y}e^{j\varphi }\right)\right]\\ cro-lin.:\sqrt{2}/2Re\left[j\cdot \left(M_{x}+M_{y}e^{j\varphi }\right)\right]\\ left\;hand\;cir.:1/2Re\left[\left(1+j\right)\cdot \left(M_{x}+M_{y}e^{j\varphi }\right)\right]\\ right\;hand\;cir.:1/2Re\left[\left(1-j\right)\cdot \left(M_{x}+M_{y}e^{j\varphi }\right)\right] \end{array}$$

In the equation, $\varphi =\varphi_{y}-\varphi_{x}$ since we are only concerned with the polarization characteristics that depend on the phase difference rather than on the phase. The results of the calculations using Equation (1) are:

(2) $$\{\begin{array}{c} co-lin.:N\;\left|cos\left(\theta \right)\right|\\ cro-lin.:N\;\left|cos\left(\frac{\pi }{2}+\theta \right)\right|\\ left\;hand\;cir.:N\;\left|cos\left(\frac{\pi }{4}+\theta \right)\right|\\ right\;hand\;cir.:N\;\left|cos\left(-\frac{\pi }{4}+\theta \right)\right| \end{array}$$

where $N=\sqrt{{\left(M_{x}+M_{y}cos\varphi \right)}^{2}+{\left(M_{y}sin\varphi \right)}^{2}}/\left(\sqrt{{\left(1+cos\varphi \right)}^{2}+sin^{2}\varphi }/\sqrt{2}\right)$. Its numerator is the modulus of $\left(M_{x}+M_{y}e^{j\varphi }\right)$; and the denominator is the modulus of $\sqrt{2}/2\left(1+e^{j\varphi }\right)$, which represents that the MS is lossless. The denominator acts as a normalization factor in order to ensure that the received manipulation no greater than 1. θ is the argument of $\left(M_{x}+M_{y}e^{j\varphi }\right)$ that can be formulated as $arctan\left(\frac{M_{y}sin\varphi }{M_{x}+M_{y}cos\varphi }\right).$ It is clear that the change in φ is crucial for the reception magnitude change. The variation of the reception of the differently polarized receivers will be the same if φ remains unchanged. The receive magnitude in the lossless case is depicted in Figure 1b, where the period is 2π for all receive polarizations. In Figure 1b, co- and cross-polarized receivers are covariant, and left- and right-circular-polarized receivers are also covariant, which means that one receiver in each pair is necessary for phase inference. Nevertheless, a certain receiver magnitude corresponds to the two phase cases in a period. Ambiguity resolution is performed by the combined use of one linear-polarized and one circular-polarized receiver.

To consider a scenario where the MS is rotated by 90°, while the incidence is unchanged, the Jones Matrix of the rotated metasurface can be denoted as $\left[\begin{array}{c} M_{y}e^{j\varphi_{y}},0\\ 0,M_{x}e^{j\varphi_{x}} \end{array}\right]$, i.e., magnitude/phase response of reflection will be transformed to $M_{y}/\varphi_{y}$ and $M_{x}/\varphi_{x}$ along the x and y axes, respectively. The results of the calculation based on Equation (1) can be written as:

(3) $$\{\begin{array}{c} co-lin.:L\;\left|cos\left(\psi \right)\right|\\ cro-lin.:L\;\left|cos\left(\frac{\pi }{2}+\psi \right)\right|\\ left\;hand\;cir.:L\;\left|cos\left(\frac{\pi }{4}+\psi \right)\right|\\ right\;hand\;cir.:L\;\left|cos\left(-\frac{\pi }{4}+\psi \right)\right| \end{array}$$

where $L=\sqrt{{\left(M_{y}+M_{x}cos\varphi \right)}^{2}+{\left(M_{x}sin\varphi \right)}^{2}}/\left(\sqrt{{\left(1+cos\varphi \right)}^{2}+sin^{2}\varphi }/\sqrt{2}\right)$, the numerator is the modulus of $\left(M_{y}+M_{x}e^{-j\varphi }\right)$, and the denominator is the modulus of $\sqrt{2}/2\left(1+e^{j\varphi }\right)$, and also acts as the normalization factor. ψ is the argument of $\left(M_{y}+M_{x}e^{-j\varphi }\right)$ that can be formulated as $arctan\left(\frac{-M_{x}sin\varphi }{M_{y}+M_{x}cos\varphi }\right).$ In the lossless case, L=N and ψ=−θ in Equations (2) and (3). The received magnitude when the MS is rotated is shown in Figure 1c, in which the two linear polarizations are kept the same as those in Figure 1b, while the two circular polarizations are exchanged. The performance of the circular-polarized receiver can be used to determine the deformation direction of the MS, achieving dual-axis strain sensing.

## 3. Strain-Sensing Design and Simulation

### 3.1. Matesurface Strain Sensor Design

A dual-axis strain sensing MS was designed on basis of the above analysis. In the proposed method, different manipulation/phase responses along the two perpendicular directions will result in different reception magnitudes of the multiply-polarized receivers. The phase is the key issue for the reception magnitude variation, while the phase of the MS is very sensitive to the MS dimensions. The interplay between these two properties provides an approach for high-sensitivity strain sensing. Different deformation caused by different stress applied on the MS along the two orthogonal axes will generate different phase responses in these directions, achieving significant reception magnitude changes with changing stress.

**Figure 2:**
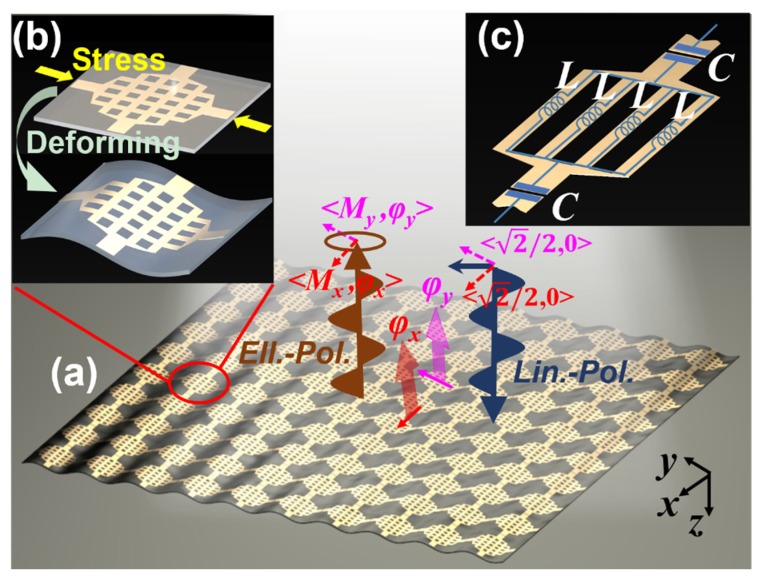
(**a**) Schematic of the wrinkled MS consisting of periodically arranged lantern-shaped elements shown in (**b**). Circuit model analysis is proposed in (**c**).

To evaluate the performance of the proposed method, a sensing system composed of a reflective phase-deformation sensitive MS, a linear-polarized incidence, and multi-polarized receivers is proposed. The schematic of the employed MS is shown in Figure 2a. The incidence is divided equally into two components along the x and y directions, respectively. After deformation, the reflective magnitude/phase from the proposed MS will be different since the deformations in these two directions are different and are equal to $M_{x}/\varphi_{x}$ and $M_{y}/\varphi_{y}$ along the x and y axes, i.e., the outgoing wave becomes elliptical-polarized. It is clear that the phase difference caused by the deformation is more noticeable than the change in the magnitude, which is the key point for the reception magnitude shift.

A flexible MS for strain sensing is also shown in Figure 2a where its unit cell is a bilayer structure with two same metallic lantern shapes cross-patterned on the top layer with a backboard on the bottom layer, as shown in Figure 2b. A stress is applied to the MS, giving rise to a sinusoidal wrinkle. Here, we assumed that the deformation period is equal to the size of a single unit cell. For industrial applications, the MS can be mechanically trained in an appropriate manner to ensure that the MS deforms with a period of multiple unit cells, as reported in [21,22,29].

To ensure the MS flexibility, the printed circuit board (PCB) used for MS fabrication should be sufficiently thin, which means the substrate is low profile. Meanwhile, the relative dielectric constant of the employed PCB must be lower than 2.6 since PCB will be hard if its dielectric constant is higher than 2.6 in fabrication. In our design, a Taconic TLY-5 substrate with a thickness of 0.254 mm, relative dielectric constant of 2.2, and loss tangent of 0.001 is used. Nevertheless, low profile and low permittivity give rise to a limit for which the phase cannot change remarkably with deformation, in particular for low profile. In our study, a lantern-shaped pattern is employed to achieve significant phase variation with the deformation due to its equivalent circuit model. As shown in Figure 2c, the total inductance is a combination of four lead inductances L in parallel, resulting in a sharp decrease of the inductance to L/4, while the length of the gap between the adjacent unit cells is decreased in order to decrease the capacitance C. The element space is 10 mm. The length and width of the gap for building C are 1.1 mm and 0.2 mm, while of the metallic line for building L are 5.8 mm and 0.5 mm. The distance between the metallic lines is 1.2 mm.

### 3.2. Sensor Simulation and Results Analysis

A model of the proposed unit cell was developed in ANSYS-HFSS using periodic boundary conditions (Master/Slave boundary) to simulate an infinite MS. In the simulations, the unit cell was sinusoidally deformed with a period of a single element under five strain states applied along the y direction while no stress was applied along the x direction. The unit cell deformation varied evenly from 1 to 5% along the y axis. It is important to note that even though the dimensions of the unit cell necessarily change with the applied strain, the side length of the unit cell was kept constant in the parameter retrieval process for all of the configurations/strain states along both x and y axes.

**Figure 3:**
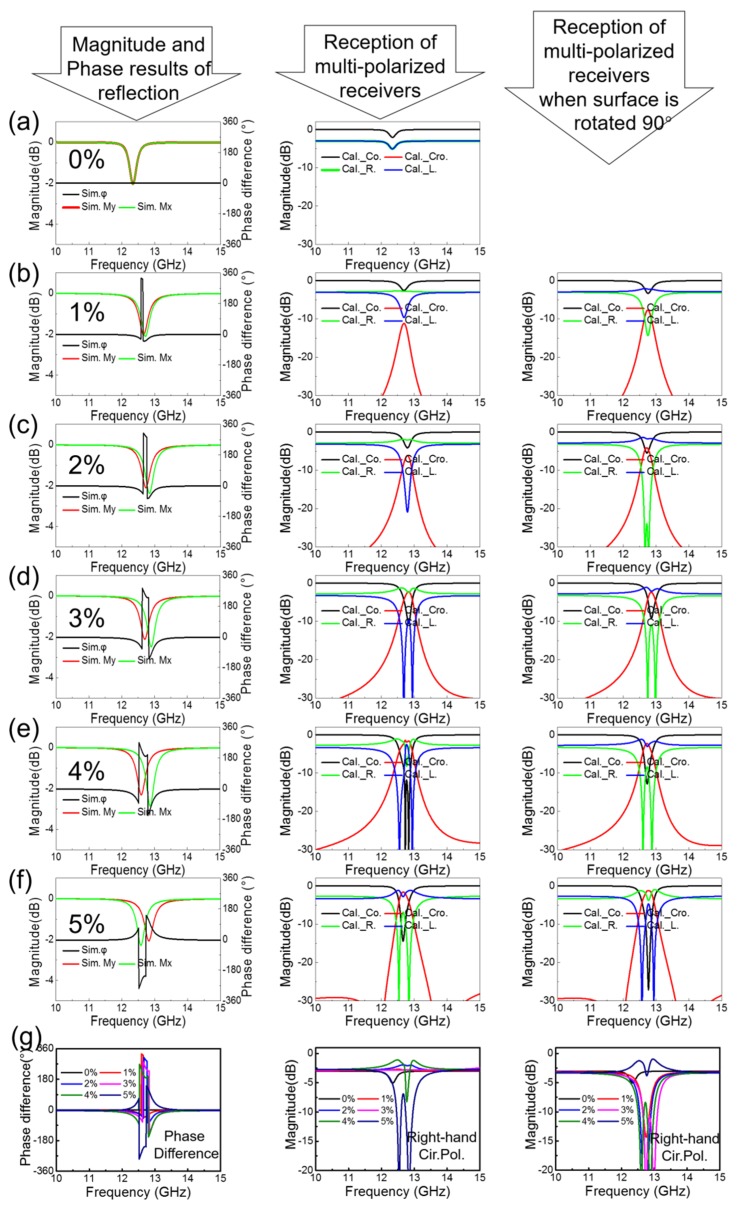
Simulation results for 0–5% strain states are shown in (**a**–**f**), respectively. The graphs in the first column show the results of the difference between the reflective phases along the y and x axes and the magnitude of the reflective components along the y and x axes. The multi-polarized reception results for the deformations along the y and x axes are depicted in the graphs presented in the second and third columns, respectively. In (**g**), comparisons of phase difference, right-hand circular-polarized reception are depicted.

The results for $M_{x}/M_{y}$ and $\varphi =\varphi_{y}-\varphi_{x}$ obtained in the simulations are depicted in the first column of Figure 3, where the 0–5% strain states are represented in Figure 3a–f, respectively. The 0% state means that no strain was applied to the MS and was used for comparison. It is observed that the magnitude/phase response of the planar MS is isotropic while the loss of the MS is low even at the operational frequency (−2 dB at 12.3 GHz). An examination of Figure 3b–f shows that the magnitude/phase responses along the x and y axes are different due to the different deformation states caused by the anisotropic applied strain. It is clear that the magnitude and operational frequency change little, whereas the phase shifts significantly. This is the fundamental basis of our method for sensitivity improvement of strain sensing. Using Equation (2), we calculate the magnitude received by the multiple-polarized receivers, with the results shown in the second column in Figure 3 for the strain cases from 0 to 5% strain in Figure 3a–f, respectively.

Analysis of these results shows that the resonance frequency was shifted independently along the x and y directions, inducing different phase response shifts in these directions, and resulting in a sharp variation in the phase difference φ under the pressure-induced deformation. The reception magnitude of the multiply-polarized receivers presented in the second column shows a sharp change with the deformation for all polarization responses. The separation between the resonant frequency in the x and y directions increases with increased deformation, resulting in a rise in the cross-polarized component and a decrease in the co-polarized component at approximately 12.7 GHz. The left-hand circular-polarized receiver shows a remarkable change for 1–4% strain states and, the right-hand circular-polarized receiver shows a remarkable change for the last strain, due to the phase difference in these two groups behaving in the opposite manner, as demonstrated in the first column. Overall, a significant variation in the receiver magnitude of the multiply-polarized receivers is obtained when the proposed MS is wrinkled by applying strains of different magnitude. Taking the cross-polarized receiver as the example, it is observed that the 1% strain led to a reception increase from −30 dB to approximately −10 dB at the operational frequency, corresponding to the improvement by a factor of 100. Moreover, the MS can be utilized to predict the deformation since the magnitude change is also distinct. Taking the variation between the 1% to 2% stain states as an example, it is observed that the cross-polarized reception shifts from −10 dB to −5 dB (a factor of approximately 3), while the left-hand circular-polarized reception shifts from −10 dB to −20 dB (a factor of approximately 10). The proposed MS strain sensor shows good performance in strain sensing. As designed, our method provides an improved approach for high-sensitivity strain sensing. The magnitude response in the frequency domain has been shown to vary sharply with the pressure-induced deformation, while for tension deformation, we can use two approaches: (i) an interim strain case such as the 2% strain can be set as the initial state; (ii), use of a stretchable substrate to replace Taconic TLY-5 will lead to the phase showing similar behavior, and will result in a distinct change of the multiply-polarized reception under tension deformation.

As mentioned above, Equation (3) describes the characteristics in the case of same deformation along the x axis, where the behavior of the linear-polarized pair will be unchanged while the polarizations of the components of the circular-polarized pair will be exchanged, as shown by the comparison of Figure 1b,c. Here, the same strain cases as those along the y axis are simulated, and the results are calculated using Equation (3) for the strains applied in the x direction. The calculation results are shown in the right-most column for the strains with the magnitudes ranging from 1% to 5% as shown in Figure 3b–f. The 0% strain is not shown because it is the same as that for the strain along the y direction due to its isotropic nature. An examination of the results shows that the linear-polarized reception is roughly the same as the corresponding results in the mid-column, while for the circular-polarized pair, the left-/right-hand receiver shows similar performance to that of the right-/left-hand receiver for which the results are shown in the middle column. Therefore, the method is verified to be capable of dual-axes sensing operation. The direction of the applied strain can be deduced by the combined analysis of the linear- and circular-polarized reception magnitudes. Comparisons of phase difference, right-hand circular-polarized reception are depicted for strain states from 0% to 5%. The phase difference is shift with states significantly. The circular-polarized reception changes a litter in some strain cases. However, the linear-polarized reception is changed a lot in these cases.

As a summary of the capability of sensitivity on basis of our method by comparing with research on characteristics of magnitude or frequency, the performance improved significantly. In previous studies, for the 1% strain state (which can be regarded as a slight strain applied), on characteristics of magnitude, the magnitude shift $\left|\frac{Magnitude\;in\;1\%\;strain\;state}{Magnitude\;at\;no\;strain\;case}-1\right|$ of concerned feature is about 25% in [28] and 10% in [27], while of frequency, the frequency shift $\left|\frac{Frequency\;in\;1\%\;strain\;state}{Frequency\;at\;no\;strain\;case}-1\right|$ is about 0.15% in [21] and 4% in [22]. By employing our method, the magnitude shift of co-polarized linear-polarized, left-hand, and right-hand circular-polarized component is around 20%, 40%, and 100%, respectively. The change of cross-polarized component is more significant by a factor of 100. The sensitivity improved markedly owing to the reflection analysis took into account the phase characteristics.

## 4. Fabrication and Measurements

**Figure 4:**
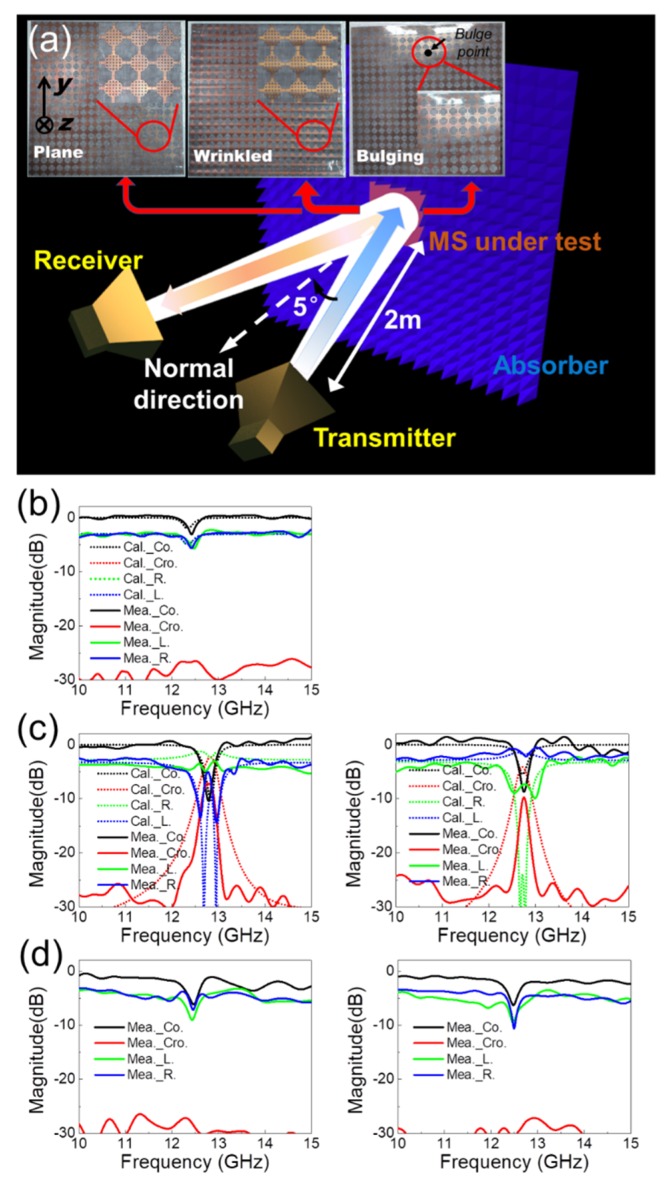
Measurement of the proposed MS. (**a**) Schematic of measurement setup for the cross linear-polarized case, and fabricated MS cases in the planar, wrinkled and bulging strain states. (**b**–**d**) show the measured results for the planar, wrinkled and bulging MS, respectively, with the corresponding simulation results depicted for comparison.

To verify the design, the proposed MS was fabricated and measured. The fabrication of MS is on basis of PCB technology, i.e., utilizing chemical etching on the metallic sheets of the substrate. Taconic TLY-5 substrate is employed in MS fabrication, of which the dielectric constant is 2.2 and the thickness is 0.254 mm. The whole MS is composed of 20 × 20 proposed lantern-shape elements. The fabrication of MS is on basis of PCB technology, i.e., utilizing chemical etching on the metallic sheets of the substrate. For achieving the deformed MS, a metallic plate whose top layer is with the desired sinusoidal shape is employed as a mold. The deformed MS can be achieved after pressing plane MS on the sinusoidal surface of the mold. Due to the limitation of our processing capabilities, a mold for the 3% strain case was fabricated as a sample MS used to verify the proposed method. Nevertheless, the mold MS did not fit the mold very well, with the deformation measured as slightly smaller than 3% after the fabrication. A case where a bulge with a size of 5 mm bulge is located at approximately one quarter of the length was also examined in order to simulate a scenario found in practical applications where the strain is only applied at this point. A schematic of the test setup of the cross-polarized reception is shown in Figure 4a, and the fabricated proposed MS in different strain cases and their magnified views are illustrated in the insets and from left to right represent the planar, 3% wrinkled, and bulging MS, respectively. The transmitter is a horn antenna polarized along the direction with a 45° angle with +x axis. The receivers are four horn antennas in different polarizations, composed of co-linear-polarized, left-hand and right-hand circular-polarized receivers to test the multi-polarized response. The angle between the propagation direction of the transmitter/receiver and MS normal is approximately 5°, simulating that the test antenna is normal incident, while the distance between the test antenna and MS is approximately 2 m. It is important to note that the measurement is calculated by a copper plane with the same dimension of the MS under test, which simulates the perfect electric conductor (PEC) plane, in the case of that the transmitter and receiver are the same polarized. The reception magnitude of MS is calculated by subtracting its response and the PEC response.

The measured results for the planar MS are shown in Figure 4b and are also compared to the results of the simulations. Figure 4c shows the measured and simulated (3%) results of the wrinkled case, where the first column denotes the deformation along the y axis while second column is for the deformation along the x axis (i.e., the strain is applied in the cross direction). The results of the bulging case are presented in the same manner for Figure 4d to simulate the strains applied in the y and x axes, respectively.

An examination of Figure 4b shows that good agreement between the simulations and measurements is obtained for all polarized receptions for the features of resonant frequency and reflection. The loss of the MS is approximately −3 dB, which means that the MS is low-loss and also implies that the response variation with deformation is mainly caused by the phase characteristics. The measured results for the wrinkled MS under the application of 3% strain are shown in Figure 4c, and it is observed that the performance for the co-linear- and two circular-polarized reception is generally in agreement with the simulation results. The receive magnitude of the cross linear-polarized component is approximately 4 dB lower than that obtained in the simulation because of (i) the relatively large test error in cross-polarization measurements, and (ii) the deformation of the MS shaped using a mold not being able to fully match the model used in the simulations. By analyzing the results of other three polarization and the loss of the MS, the magnitude of the experimental cross-polarized component can be improved to match the simulation results. In addition, the linear-polarized components are found to be roughly the same after rotating the deformation direction of MS from the y to the x axis, while the polarizations of the two circular-polarized components were exchanged, validating the method and the simulations. The curves presented in Figure 4c for the bulging MS case showed similar trends to the results for the planar MS presented in Figure 4b. Nevertheless, the reception magnitude is lower than that in Figure 4b, while the difference between the magnitudes increases with increasing frequency. This is because the bulge of MS leads to the change of the radiation direction of reflection from the mirrored direction of incidence. Since the directionality of the test antenna is higher at a higher frequency, the reduction in the reception increased.

## 5. Conclusions

In conclusion, this work presents a method for MS-based strain sensing. Using this approach, the sensitivity can be improved significantly because the phase of the reflection from MS shifts sharply with deformation. We first determined the relationship between the multi-polarized receptions and anisotropy phase shift to be used as the basis of an alternative and feasible approach for phase measurement. To verify the proposed method, a flexible MS that showed a significant phase change with deformation was used based on the lantern-shaped unit cell design. In the simulations, the proposed MS showed good performance in strain sensing for the strain states in the 1–5% strain range. The MS was fabricated and three cases of the planar, wrinkled, and bulging MS were evaluated, with the obtained test results in good agreement with the predictions of the simulations for the designed method. This approach can also potentially be utilized in tension sensing by using a stretchable MS. The operational frequency can be improved to THz and even to the optical band because the mathematical analysis that provides the basis of this method is valid for all EM radiation frequencies.
